# App-based strength and balance self-test in older adults: an exploratory study from a user perspective

**DOI:** 10.1186/s13104-021-05792-5

**Published:** 2021-09-26

**Authors:** Marina Arkkukangas

**Affiliations:** 1 Research and Development in Sörmland, Region Sörmland, Eskilstuna, Sweden; 2 School of Health and Social Studies, Department of Medicine, Sport and Fitness Science, Dalarna University, Falun, Sweden; 3 Department of Physiotherapy, School of Health, Care and Social Welfare, Mälardalen University, Västerås, Sweden

**Keywords:** Strength, Balance, Self-assessment, Older population, Smartphone application

## Abstract

**Objectives:**

Falls are a common problem, especially in the older population. The number of older adults aged over 65 years is increasing globally, leading to a major challenge in providing effective fall prevention interventions to older adults requiring such interventions. This study aimed to explore the usability of an app-based strength and balance self-tests in a small sample of four older adults. This study is a side product of another project.

**Results:**

The results from this study indicated that self-test of strength and balance by using a smartphone application is a challenge for older adults. Basic test measures, such as start and stop and counts of sit-to-stand, were difficult to self-administer. However, from a user perspective, the possibility of independently performing these measures was considered important and needed to be further developed and evaluated in future studies.

## Introduction

Falls are a common problem, especially in older adults, with over 30% of community-dwelling older adults experiencing one or more falls annually and the risk steadily increasing with age. Falls are the leading cause of disability and accidental death in older adults [1, 2]. Several functions are closely associated with risk for falls, especially strength and balance functions, highlighted and well documented in the fall prevention research literature [3, 4]. Objective measures of strength and balance in older adults are widely used to predict falls, and detecting changes at an early stage could be crucial to improving or preventing future falls [5]. Clinical instruments have been developed to address strength [6] and aspects of balance [7]*.* However, these clinical instruments are predominantly used by professionals and not for self- tests. Recently using smartphones to assess strength and balance has shown promising results and correlations with commonly used clinical instruments [8–10]. Further digitally provided fall prevention exercises and self-assessment of physical functions via smartphones or personal computers have previously been investigated. There is great potential for using smartphones as a tool for self-assessing physical functions [11, 12]. These innovations in mobile health (mHealth) technology provide new possibilities in assessing older adults’ strength and balance functions.

In 2017, a research project started in Sweden aiming to develop a fall prevention exercise application for smartphones [13]. The application included exercises from one of the most commonly used fall prevention programs, the Otago Exercise Programme (OEP) [14]. During the application’s development, an interest in adding fall risk self-assessment emerged, which was further investigated as a side product of the main project. Since two common strength and balance clinical tests are used before fall prevention exercise prescription, these were of interest to further investigate and to integrate in the application. This study aimed to explore usability of app–based self-tests of strength and balance functions in a small sample of four older adults.

## Main text

### Methods

In this study, three women and one man, aged 68–83 years, participated. These four older adults were recruited from an ongoing project evaluating the usability of an exercise application based on OEP [13]. They were all recruited by contact with an organization for retired persons in middle Sweden. In a monthly organization meeting, the prospective participants received verbal and written information about the purpose of the study. They were invited to participate in the development process of evaluating the usability of the app-based fall prevention exercise by using a smartphone (Android). Therefore, they were all familiar and had experienced using smartphones before this study. The choice to use a smartphone application was made since smartphones are also increasing among older adults. The target group in the main study was age ≥ 65 years, able to walk independently, and without any cognitive impairments. In this study, two commonly used clinical tests were used when screening strength and balance functions prior to fall prevention exercise prescription. The tests were developed as standalone tests and integrated into the fall prevention exercise application under development. The test used was;

*Chair stand test *(*CST*), a chair is placed against a wall, and sit-to-stand is performed five times as fast as possible while timed. Performing five sit-to-stands for more than 15 s has shown an increased risk of falling [6].

*Four-test balance scale* consists of four static balance positions that increase in difficulty and must be performed without support; feet together, semi-tandem stance, tandem stance, and one-leg stand. Each position should be held for 10 s without moving the feet or needing support. If the person does not stand in a tandem position for 10 s, there is a risk of falling [7].

For this study, a prototype was developed for pilot testing in five healthy middle-aged individuals before the test in older adults as a safety precaution before introducing the app to the older participants.

#### Description of app-based tests

**Fig. 1:**
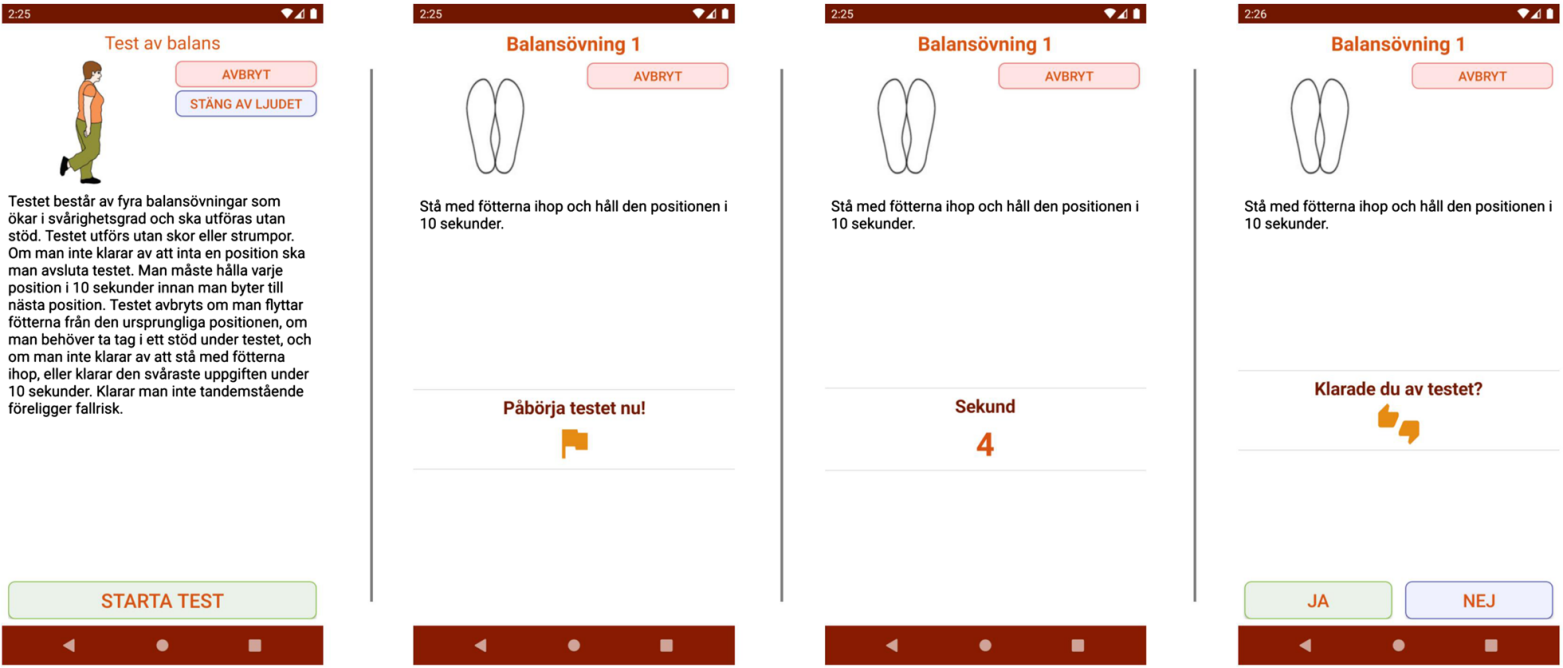
Screenshots of four-test balance scale

For both tests, illustrations, text, and voiceover instruction of the performances (including timing) are presented in all steps (see Fig. 1). Each test started with instructions to take the position and, when ready, click the start button to initiate the test.

For the four-test balance scale, the time stops automatically after 10 s, and participants press “thumbs up or thumbs down” to manage the test and move forward to the next step (Fig. 1). The CST has the same structure as the four-test balance scale, except for the timing of the performance, which must be stopped manually after five sit-to-stands.

#### Procedure

Before testing, a chair was placed in the room for the CST test. The participants were asked to self-administer the tests using the app on their smartphones. The participants did not receive any guidance from the assessors during the tests. Two assessors, one physiotherapist (PT) and one research assistant were present during the tests. The assessors observed while the participants attempted to self-administer the tests, recording issues and errors using field notes.

The participants were unfamiliar with the tests before the session. The participants were told to hold the smartphone in their hands during the performance. Instructions were provided in the application, text, visual, and voiceover that instructed the user on what to do once the test sequence had been initiated. After the participants had performed the tests, the PT performed the test according to the original clinical test manual.

### Results

The participants completed the tests, and the results from each test are presented in Table 1. Each session lasted approximately 30 min.

**Table 1:** Results of the self-test accompanied by a clinical test (assessor) (n = 4)

Observation NR 1 (female)	
*TEST 1—Strength (self-test)*	*TEST 1—Balance (self-test)*
• Unclear when to press the start button • Started the test before the instructions were completed • Needed help to stop the time when finished with five sit to stand	1. Completed stage 1 2. Completed stage 2 3. The test was disrupted since the participant needed support at stage 3 4. X
Results: 10 s	Results: Completed stage 1–2
*TEST 2—Strength (clinical test)*	*TEST 2—Balance (clinical test)*
Instructions were followed according to the manual	Instructions were followed according to the manual (no support)
Results: 8.09 s	Results: Completed the test

Observation NR 2 (female)	
*TEST 1—Strength (self-test)*	*TEST 1—Balance (self-test)*
• Followed the instructions correctly • Developed insecurity in counting sit-to-stand five times, lost track of counts	1. The feet were not close together in stage 1, completed stage 1 2. Standing on one leg, which was incorrect at the second stage, she corrected her mistake, completed stage 2 3. Correct performance, completed stage 3 4. Correct performance, completed stage 4
Results: 26 s	Results: Completed the test
TEST 2—Strength (clinical test)	TEST 2—Balance (clinical test)
• Instructions were followed according to the manual	• Instructions were followed according to the manual
Results: 10.61 s	Results: Completed the test

Observation NR 3 (male)	
*TEST 1—Strength (self-test)*	*TEST 1—Balance (self-test)*
• Listened carefully • Performed the test relatively early according to instructions • Lost count of sit-to-stand	1. Completed stage 1 2. Completed stage 2 3. Completed stage 3. Relatively performed swaying 4. Completed stage 4. Started performing the last test relatively early
Results: 23 s	Results: Completed the test
*TEST 2—Strength (clinical test)*	*TEST 2—Balance (clinical test)*
Performed the test according to the instructions	Performed the test according to the instructions
Results: 15.33 s	Results: Completed the test

Observation NR 4 (female)	
*TEST 1—Strength (self-test)*	*TEST 1—Balance (self-test)*
• Performed the test according to the instructions • Perfectly performed the start and stop	1. Completed stage 1 2. Completed stage 2 3. Completed stage 3 4. Completed stage 4. Relatively performed swaying. Started the test relatively early
Results: 16 s	Results: Completed the test
*TEST 2—Strength (clinical test)*	*TEST 2—Balance (clinical test)*
• Performed the test according to the instructions	• Performed the test according to the instructions. Lost balance at stage 4, managed 7,79 s
Results: 10.46	Results: completed stages 1–3

#### Field notes

The participants expressed that the tests were significantly interesting to perform; they were also significantly satisfied with their results. The tests were difficult to perform in the beginning since their bodies were relatively stiff at first. Subsequently, their bodies were more flexible, and the test was more easily performed. They also expressed that these measures should be performed at the same timepoint to achieve more exact performance each time.

The participants expressed that they were relatively “sloppy” when they performed the tests. The instructions were the most important part. All instructions needed to be significantly thoroughly described, and the participants should have sufficient patience to listen fully to the instructions before performing the tests. In the CST test, the voiceover of the counts of seconds was confusing, and they lost track of their sit-to-stand counts. The participants also believed that the shortcoming of counts sit to stand would be overcome if tests were automatically registering movements from sit to stand. According to the participants, they also missed pressing the button when the tests were completed. This needs to be clear since the results otherwise will be inaccurate.

In the four-test balance scale, the participants were not familiar with the word semi-tandem, even if an illustration accompanied the word. Overall, the start and stop buttons need to be more visible by using strong colors.

### Discussion

This paper describes the usability testing of two commonly used clinical tests for identifying fall risk among older adults, CST and the four-test balance scale. One common error in both tests was to press “Start test” without first observing and listening to the instructions. Further error was to press “Stop test” in CST. These usability issues were also confirmed in previous research when performing self-test of physical functions in older adults [8, 12]. These basic test measures, such as start and stop and sit-to-stand counts, significantly affect the results (time). However, smartphones have been suggested to be capable of becoming a widespread and low-cost tool for assessing balance and physical functions; therefore need to be further evaluated and validated [8, 15]. From a user perspective, the participants expressed the possibility of independently performing these measures as important, positively influencing motivation to perform these basic test measures and acquire knowledge regarding their health status. The self-test and clinical test differed in both time and performance quality, which were previously addressed in a study by Mansson et al. [16]. They concluded balance performance to be a challenge to measure, including increased postural sway, which was observed in this study during the self-test. This aspect is especially important in practice when adjusting and prescribing correct individual balance exercises, emphasizing the risk of falls.

The user perspective is important when designing and developing new ways to measure parameters important to health, especially for self-test purposes. Therefore, the gap between functional evaluation and user experience with technology is addressed in this study and highlighted in a systematic review by Ruopeng and Sosnoff [17]. There is a need to include the users in the development process, and in this study, participants’ performance was valuable for further development of the application in the main project.

This study indicated that performing self-tests is considered a challenge for older adults to self-administer correctly. There is a need to explore ways to use technology appropriately to self-assess physical functions, such as strength and balance, safely and purposefully aiming towards independence in older adults.

### Conclusion

The results from this study suggest smartphones as a future alternative for self-assessment of strength and balance in a non-supervised setting. There is a need to develop tools that are easy to use, easy to understand, have clear instructions, visually guide users, and have some automatic functions to support the performance when self-administrating strength and balance functions. Validity between app-based self-tests and clinical instruments for strength and balance needs to be further validated in future studies.

## Limitations

This study has some limitations. The sample size was small, comprising four participants only. However, the experiences and results obtained from this study were consistent and provided valuable information to be included in the further development of self-tests in fall prevention. The field notes could be viewed as a limitation; however, this pragmatic way of collecting data was satisfactory and provided valuable knowledge in our study for further development.

## Data Availability

The software and code are not available, other app related material and datasets used and/or analysed during the current study are available upon reasonable request to the corresponding author.

## Abbreviations

OEP: Otago Exercise Programme
PT: Physiotherapist
